# Severe Life Threatening Maxillofacial Infection in Pregnancy Presented as Ludwig's Angina

**DOI:** 10.1155/IDOG/2006/51931

**Published:** 2006-08-17

**Authors:** Shelly Abramowicz, Jacques S. Abramowicz, M. Franklin Dolwick

**Affiliations:** ^1^Department of Oral and Maxillofacial Surgery, University of Florida School of Dentistry, Gainesville, FL 32610, USA; ^2^Department of Obstetrics and Gynecology, Rush University, Chicago, IL 60612, USA

## Abstract

*Background*. Ludwig's angina is a rapidly spreading cellulitis that may produce upper airway obstruction often leading to death. There is very little published information regarding this condition in the pregnant patient. *Case*. A 24-year old black female was admitted at 26 weeks gestation with tooth pain, submandibular swelling, severe trismus, and dysphagea, consistent with Ludwig's angina. Her treatment included emergent tracheostomy, incision and drainage of associated spaces, teeth extraction, and antibiotic therapy. *Conclusions*. During a life threatening infectious situation such as the one described, risks of maternal and fetal morbidity include both septicemia and asphyxia. Furthermore, the healthcare provider must consider the risks that the condition and the possible treatments may cause the mother and her unborn child.

## INTRODUCTION

Ludwig's angina, named after the German physician who described the condition for the first time in 1948, is a rapidly spreading cellulitis that may produce upper airway obstruction often leading to death. The most common cause of Ludwig's angina is an odontogenic infection, from one or more grossly decayed, infected teeth, and is usually as a result of native oral streptococci or a mixed aerobic-anaerobic oral flora [1]. Additional possible etiological factors include sialadenitis, compound mandibular fractures, or puncture wounds of the floor of the mouth [1]. While this is a life threatening infection, an extensive literature search did not yield much published information regarding this condition in the pregnant patient.

## CASE REPORT

**Figure 1:**
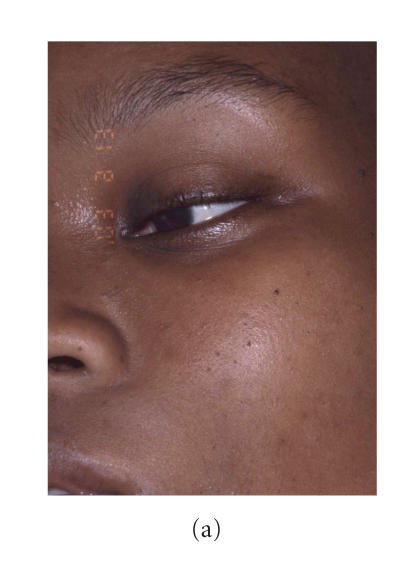

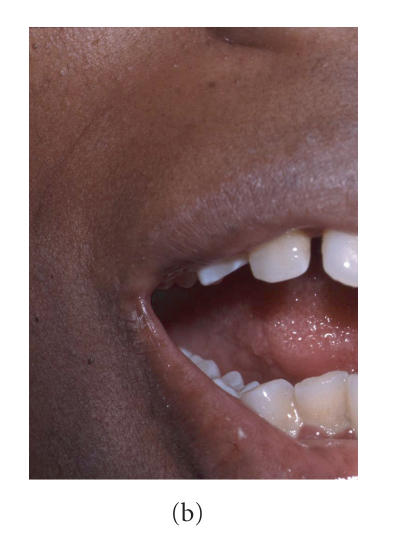
Patient at presentation. (a) General view of the face. Note severe submental and upper neck swellings. (b) Trismus: this is the maximal mouth opening.

**Figure 2:**
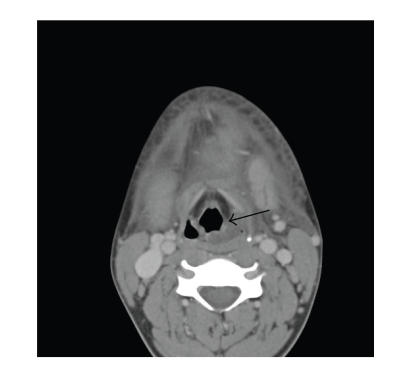
CT-scan demonstrating fluid collections in the left lateral pharyngeal space (arrow).

**Figure 3:**
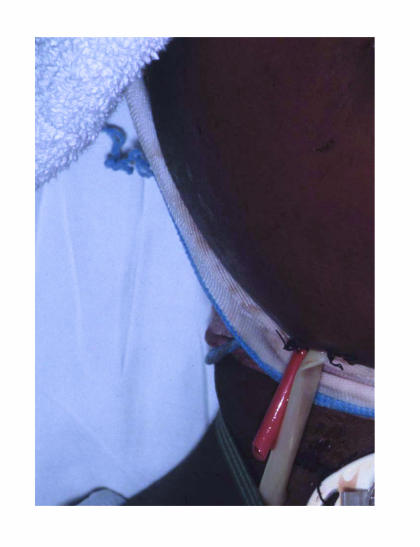
Patient postintervention. Note large number of drains.

A 24-year old black female, G4P3, at 29 weeks gestation, was presented to a local hospital complaining of facial swelling (Figure 1(a)). The patient's only significant medical history included history of anemia and sickle cell trait. She described that her pregnancy had been proceeding without difficulty, except for a three-day history of lower left quadrant tooth pain, and a one-day history of fever and chills. On presentation, her vital signs were the following: temperature 38.5°C, blood pressure 115/44, pulse 118/min, respiratory rate 18/min, oxygen saturation on room air 96%, and white cell count of 16800/μL. Her clinical presentation included large soft tissue swelling under her mandible, extending bilaterally to the angles of the mandible and inferiorly approximately down to her hyoid bone. Because of her severe impending airway deterioration, she was flown by helicopter to a tertiary medical center for definitive care. In the Emergency Room of the University of Florida Shands Hospital, the diagnosis of Ludwig's angina was made. It was difficult to perform an adequate oral exam secondary to pain, swelling, and severe trismus (Figure 1(b)) which allowed her to open her mouth to only 15 mm (average range 40–45 mm). The patient was having difficulty maintaining her own salivary secretions because of dysphagea but denied dyspnea. Since in similar situations patients can desaturate very quickly, even though her oxygen saturation was recorded to be 96% on room air, she was given supplemental oxygen and a pulse oxymeter was placed on her right index finger because of possible impending quick respiratory difficulties. Of note, although not used, an emergent cricothyrotomy kit was available at the patient's bedside at all times. The Department of Oral and Maxillofacial Surgery (OMFS) formally consulted the Departments of Obstetrics and Gynecology (Ob/Gyn) and Anesthesiology. Understanding that in administering medications and/or undergoing any surgical treatment in pregnancy, one must consider the risks and the benefits both to the mother and the unborn, it was determined that the benefits of proceeding with emergent and immediate surgical intervention outweighed the risks. An emergent CT-scan showed fluid collections in the left lateral pharyngeal space (Figure 2) extending to the level of the valleculae. Securing an airway via an awake fiberoptic nasal intubation was risky: a fiberoptic tube inserted into the pharynx might puncture an abscess and cause pus aspiration or swallowing. Therefore, an awake tracheostomy was performed using a 60-nonfenestrated Schiley endotracheal tube. At that time general anesthesia was administered and the patient was placed in slight left lateral decubitus position to decrease uterine aortocaval compression pressure. After usual sterile preparation, lidocaine with epinephrine was infiltrated for local anesthesia. An incision was made at the submental and bilateral submandibular areas and blunt dissection to the lingual inferior border of the mandible was carried out. Through the submental incision, the blunt dissection continued through the mylohyoid muscle to the sublingual areas to access all abscesses. Tooth number 17 (lower left third molar) was then extracted since it was believed that this grossly carious and partially impacted tooth was the primary source for the infection. Upon removal, purulence was expressed through the extraction socket. Five additional grossly carious teeth were then extracted. Intraoral blunt dissection to gain access into the lateral pharyngeal space followed for drainage of another pus collection. Numerous Penrose drains and red rubber catheter drains were left in place to maintain drainage of pus and facilitate daily irrigation (Figure 3). Upon admission to the Surgical Intensive Care Unit, the Department of Ob/Gyn continued following up on the patient by frequent monitoring of the fetal heart rate. The patient was extubated after 6 days and remained in the hospital until discharge the following day. On discharge, the patient's progress was monitored by the Departments of OMFS and Ob/Gyn.

## DISCUSSION

The unique anatomy of the floor of the mouth plays an important role in the development and extension of intraoral infections. The usual infectious course begins with a periapical dental abscess of the second or third mandibular molar. The roots of these teeth extend inferior to the insertion of the mylohyoid muscle, so that if untreated, the infection may continue from primary spaces to penetrate the thin inner cortex of the mandible and will involve the posterior margin of the mylohyoid muscle to the submandibular space [2]. At this time, the infection may develop and progress at such an alarming rate that special precautions regarding airway maintenance must be taken. Because the mandible, hyoid bone, and superficial layer of the deep cervical fascia limit tissue expansion associated with the developing edema, the floor of the mouth and the tongue base will become displaced superiorly and posteriorly, resulting in severe airway compromise [2]. Further extension of the infection may spread into the mediastinum and the carotid sheath resulting in severe thoracal infection. Rupture of abscesses along the way may cause aspiration of pus into the lungs and/or even pericarditis. Untreated, the mortality is close to 100 percent, both from the acute sepsis and from airway obstruction [1]. The patient with Ludwig's angina will have severe and obvious extraoral swellings including bilateral submandibular, submental, and sublingual spaces. Common presentation is elevation and displacement of the tongue, trismus, drooling of saliva, airway obstruction, dysphagea and/or dyspnea, and a hoarse (“hot potato”) voice. With extensive use of antibiotics, most facial infections improve before they have a chance to progress to Ludwig's angina. The mortality rate from Ludwig's angina, when recognized, has decreased from 50 to 5 percent [1]. Therapy also includes early surgical removal of the source of infection (which is often grossly carious dentition) via extraction, aggressive, and vigorous incision and drainage procedures with appropriate placement of drains, along with intense and prolonged antibiotic therapy and maintenance of a patient airway. While penicillin administered intravenously and in high doses is the empirical antibiotic of choice, it is often recommended to use metronidazole as well. For patients who have had repeated episodes of dental infections, clindamycin is often the antibiotic of choice [1].

Each year it is estimated that about 50,000 women undergo anesthesia and a surgical intervention at some time during gestation for indications unrelated to the pregnancy [3]. In such situations, when medical and surgical treatments for pregnant women are considered, both the physiologic changes of pregnancy and the perinatal effects of the treatment must be considered [4]. Pregnancy is accompanied by many physiological changes which place the mother at a higher risk of infection or of doing worse once infected. First, the immune response is greatly diminished during pregnancy, thus resulting in potential faster progression of an infection. In addition, there is decreased neutrophil chemotaxis, cell mediated immunity, and natural killer cell activity [5, 6]. Moreover, approximately 50% of pregnant women complain of dyspnea by 19 weeks gestation [5] and there is some depletion in the oxygen reserve of the gravid patient. This results in lower oxygen reserve which could increase fetal hypoxia during periods of hypoventilation [4]. From an oral perspective, as pregnancy associated hormonal changes begin to affect a woman's body, the gingival tissues are affected as well. They become much more sensitive and thus susceptible to irritation from soft plaque. The plaque accumulates, becomes hard calculus deposits on the teeth, and harbors bacteria in large numbers resulting in a constant, low-grade intraoral infection. An exaggerated local inflammatory response can then begin and may result in erythematous and edematous swelling of the gingiva between the teeth, also known as pregnancy gingivitis. Approximately 70% of pregnant women have this condition, even with routine oral care [7]. This condition may be slightly painful and also bleeds easily upon routine tooth brushing. Maternal infective processes sustained especially by gram negative anaerobic bacteria, such as those leading to Ludwig's angina, have been demonstrated to cause physiologic imbalance through inflammatory cytokine production, sometimes resulting in preterm labor, preterm premature rupture membranes, and low birth weight [8, 9]. During pregnancy, women tend to maintain frequent meals and snacks, which cause further plaque accumulation, as well as an increase in decay or rapid progression of previously present decay. Because a pregnant patient has increased demands on her organs, there is increased potential for poor oxygenation. On the other hand, poor oxygenation is compromising to the fetus. An infection in itself can at times infect the placenta, uterus, and possibly the fetus, causing fetal septicemia. Treatments such as prolonged intubation and certain intravenous medications can also harm the fetus. During a life threatening infectious situation such as the one described, the risk of maternal and fetal morbidity may overshadow potential teratogenic side effects [10].

In order to prevent a similar life-threatening emergency, health care providers should not neglect even minimal complaints of dental pain. Often, if a problem is identified during the early stages of pregnancy, routine dental care can be planned to control active disease or eliminate potential problems that could increase in severity later in the pregnancy. An appropriate time for dental care from a medical standpoint is the second trimester and pregnant women usually experience the greatest sense of well being during that time [6]. Dental treatments including routine cleanings, fillings, crowns, extractions, gum treatment, and continuation of orthodontic treatment can all be provided. Dental anesthetics such as lidocaine can penetrate the placenta but, in general, do not reach it because they are used locally and in small dosages during routine dental procedures [7]. Antibiotics that are acceptable include penicillin, amoxicillin, and clindamycin. Tetracycline should be avoided since it tends to cause permanent discoloration of primary and temporary dentition of the unborn child [6]. To decrease dental pain, narcotics should be avoided as well as over the counter medications such as aspirin, ibuprofen, and related products because of the potential to affect bleeding. Morphine appears to be a safe analgesic when administered for short periods of time [7]. Ludwig's angina is life threatening because of both septicemia and asphyxia. Furthermore, in pregnancy, the risks that both the condition and the possible therapies may cause the mother and her unborn child must be considered as well as the possible consequences of the condition and therapies to both.
